# Early reduction in unplanned healthcare utilization following vagus nerve stimulation for pediatric epilepsy

**DOI:** 10.1007/s00381-026-07245-5

**Published:** 2026-05-08

**Authors:** Rakesh A. Murugesan, Samuel B. Tomlinson, Susan E. Melamed, Kathleen Galligan, Benjamin C. Kennedy

**Affiliations:** 1 Division of Neurosurgery, Children’s Hospital of Philadelphia, Philadelphia, PA 19104 USA; 2 Department of Neurosurgery, Perelman School of Medicine, University of Pennsylvania, Philadelphia, PA 19104 USA; 3 Division of Neurology, Children’s Hospital of Philadelphia, Philadelphia, PA 19104 USA

**Keywords:** Vagus nerve stimulation, Healthcare utilization, Surgical outcome, Healthcare quality

## Abstract

**Objective:**

Vagus nerve stimulation (VNS) is an established adjunctive therapy for pediatric drug-resistant epilepsy (DRE), but objective evidence of its early impact on overall health is limited. Subjective seizure diaries can be influenced by placebo effects, whereas healthcare utilization may serve as a more objective window into the early post-operative course. This study aims to evaluate healthcare utilization in the immediate and subacute recovery phase (0–3 months) following VNS implantation in a large cohort of children with DRE.

**Methods:**

We performed a retrospective, single-center, single-surgeon study of 200 consecutive patients (< 20 years old) with DRE who underwent first-time VNS implantation at a Level IV pediatric epilepsy center. The primary outcome was change in acute healthcare utilization, assessed by comparing rates of all-cause and seizure-related unplanned hospital admissions and emergency room (ER) visits in the 90 days before and after surgery.

**Results:**

Unplanned acute healthcare utilization significantly decreased in the 90-day post-operative window compared to preoperative baseline. All-cause unplanned hospital admissions decreased significantly (*p* = 0.045). This change was driven by a marked reduction in unplanned seizure-related admissions. Unplanned seizure-related admissions decreased from 34 events to 14 (58.8% reduction), and seizure-related ER visits fell from 60 to 35 (41.7% reduction). The acute surgical safety profile was excellent. Ambulatory concerns expressed by patients and caregivers (electronic, telephone, or in clinic encounters) were rare but most commonly involved pain, hoarseness, cough, and wound care questions.

**Conclusions:**

In a large, consecutive, single-surgeon series, pediatric VNS implantation was associated with an early reduction in unplanned acute healthcare utilization and an exceptional surgical safety profile. Our observations suggest that VNS provides a rapid and clinically significant reduction in unplanned seizure-related hospital admissions and ER visits within the first 3 months of implantation. This suggests an early benefit of VNS for children with DRE.

**Supplementary Information:**

The online version contains supplementary material available at 10.1007/s00381-026-07245-5.

## Introduction

Over the past three decades, vagus nerve stimulation (VNS) has become an established adjunctive therapy for drug-resistant epilepsy (DRE), including in pediatric patients [1]. The long-term benefits of VNS for children with DRE are increasingly well-established in the literature, including in large meta-analyses and randomized controlled trials [2–4]. Chronic VNS therapy is associated with reduced seizure frequency, improved neurodevelopmental trajectories, and enhanced quality of life in children [5, 6]. VNS settings are usually gradually increased over the course of weeks or months to reach “therapeutic” stimulation parameters, and it is not known whether there is an effect of VNS therapy on seizure frequency or severity during this programming process.

Although VNS therapy has some potential long-term adverse sequelae (e.g., implant-site discomfort, cosmetic concerns, device malfunction necessitating revision, etc.), much of the procedure-related morbidity accrues during the early post-operative period. Common early complications of VNS device placement include stimulation side-effects, hoarseness, vocal dysfunction, dysphagia, seroma/hematoma, and wound healing issues, among others [7, 8]. A comprehensive account of the risk-benefit profile of VNS in children requires understanding how post-procedural morbidity relates to healthcare utilization and engagement in the acute recovery phase.

Early healthcare utilization including unplanned inpatient admissions, emergency room (ER) visits, and patient communications (electronic and telephone) provide key insights into the early post-operative course following VNS placement. In this study, we comprehensively assessed early healthcare utilization in a large, consecutive, single-surgeon, single-center series of pediatric VNS cases.

## Methods

### Patient cohort

We retrospectively analyzed a consecutive, single-center, single-surgeon series of 200 patients (< 20 years old) who underwent index (i.e., first-time) VNS implantation for DRE, beginning at the start of the senior author’s career (BCK) in August 2017 to January 2025 at the Children’s Hospital of Philadelphia (Table 1).

**Table 1:** Clinical details of study population (*N* = 200)

**Age at surgery (years)**	*N* (%)
< 4	19 (9.5%)
4–12	107 (53.5%)
13–19	74 (37.0%)
**Sex**	
Male	112 (56.0%)
Female	88 (44.0%)
**Ethnicity**	
White	122 (61.0%)
Black	16 (8.0%)
Hispanic	19 (9.5%)
Asian	6 (3.0%)
Multi-racial	6 (3.0%)
Other	28 (14.0%)
Unknown/refused	3 (1.5%)
**Weight at surgery (kg)**	
< 20	42 (21.0%)
20–50	108 (54.0%)
50–100	48 (24.0%)
> 100	2 (1.0%)
**Other**	
Prior tracheostomy	6 (3.0%)

Inclusion criteria were (1) age < 20 years at the time of surgery, (2) diagnosis of DRE, and (3) first-time VNS implantation. Exclusion criteria were (1) patients undergoing VNS generator replacement or revision, (2) prior history of resective or disconnective epilepsy surgery, or (3) incomplete medical records for the 90-day pre-operative and post-operative periods.

The study was approved by the institutional review board with a waiver of informed consent for retrospective electronic record review. Standard protocols at our institution include surgical referral by the patient’s primary epileptologist, pre-surgical assessment by the neurosurgical team, and device implantation (outpatient surgery) followed by six device programming visits (~ 2 weeks apart) beginning 2 weeks after surgery. Additionally, patients were evaluated in clinic post-operatively by the neurology and neurosurgery teams within 1 month of surgery.

### Surgical procedure

All patients underwent left-sided approach for VNS device placement (LivaNova USA, Inc; Houston, TX) via a standard technique. The procedure began with a small transverse incision within a skin crease of the neck followed by subcutaneous dissection, division of platysma, and careful dissection of the carotid sheath to expose and circumferentially release a segment of vagus nerve to accommodate the device coils. The generator was placed above the pectoralis fascia through an incision in the deltopectoral groove to avoid tunneled leads crossing beneath the incision. A tension relief loop was fashioned in the neck to prevent lead traction. Vancomycin-saline irrigation was used throughout the case. Incisions were closed in standard multi-layered fashion with absorbable suture including subcuticular layer and skin glue. Patients were discharged on the day of surgery.

### Programming

Our standard protocol is for patients to return to the clinic 2–3 weeks after implantation for device activation. The initial goal for all patients was to increase current amplitude by 0.25 milliamps (mA) every 2 weeks until an output current of 1.5 to 1.75 mA was achieved. For some patients, side effects such as coughing or discomfort led to slower rates of titration, or at times, maintenance at lower settings. The AutoStimulator function was enabled. Duty cycle changes were typically not made during the 3-month post-operative period.

### Acute-care encounters

**Fig. 1:**
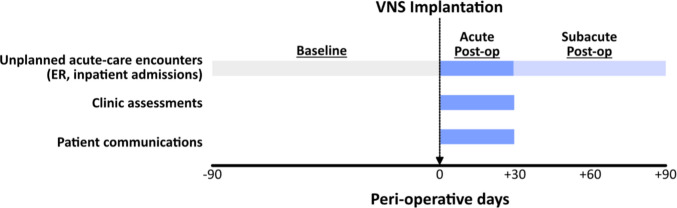
Retrospective analysis of unplanned acute healthcare utilization and ambulatory concerns. Rates of unplanned hospitalizations and emergency room (ER) visits were compared between the baseline (−90:0 days), acute post-operative (0:30 days), and subacute post-operative (30:90 days) windows of interest. All patient communications documented in the medical record (telephone and electronic), as well as all ambulatory clinic assessments, within 30 days of surgery were thoroughly reviewed for procedure-related concerns

 We retrospectively examined all documented acute-care encounters (ER and inpatient admissions) in three peri-operative time windows of interest: pre-operative baseline (−90:0 days before surgery), acute post-operative (0–30 days), and subacute post-operative (30–90 days) (Fig. 1). Encounters were manually classified as “planned” or “unplanned” based on detailed review of the clinical indication and chief complaint.

Planned encounters were excluded from the primary analysis. These included any admission scheduled in advance, such as elective procedures (i.e., dental procedures) or long-term video EEG monitoring. Unplanned encounters were defined as any ER visit or unscheduled inpatient admission. The clinical reasons for all unplanned encounters were extracted and categorized (Supplemental Figures S1, S2). Rates of acute-care encounters (events/patient-month) were calculated in each window.

### Clinic assessments and patient communications

We comprehensively reviewed all clinic encounters and patient communications within the acute post-operative period (0–30 days) to characterize the relative frequency of procedure-related concerns raised by patients and caregivers (Fig. 1). For all ambulatory encounters with neurology and neurosurgery teams occurring within 30 days of surgery, we reviewed provider notes and identified any documented procedure-related complaints or concerns expressed by the patient or caregivers. Similarly, we identified all documented telephone and electronic communications between patients or caregivers and healthcare providers within the acute 30-day window and extracted any procedure-related concerns.

### Statistical analysis

Rates of unplanned acute-care encounters were compared between baseline (−90:0 days), acute post-operative (0:30 days), and subacute post-operative (30:90 days) windows using paired one-sided Poisson tests (alpha < 0.05). This analysis was performed both for all-cause chief complaints as well as for the specific indication of unplanned seizure-related presentations. All statistical analyses were conducted using RStudio. For clinic assessments and patient communications within the acute post-operative window, we identified procedure-related complaints and summarized the frequency of each complaint descriptively.

## Results

### Clinical characteristics

A total of 200 consecutive patients who underwent first-time VNS device implantation for DRE were included (median age: 10.4 years, range 1.4–19.8 years; 56.0% male).

### Unplanned hospital admissions

**Fig. 2:**
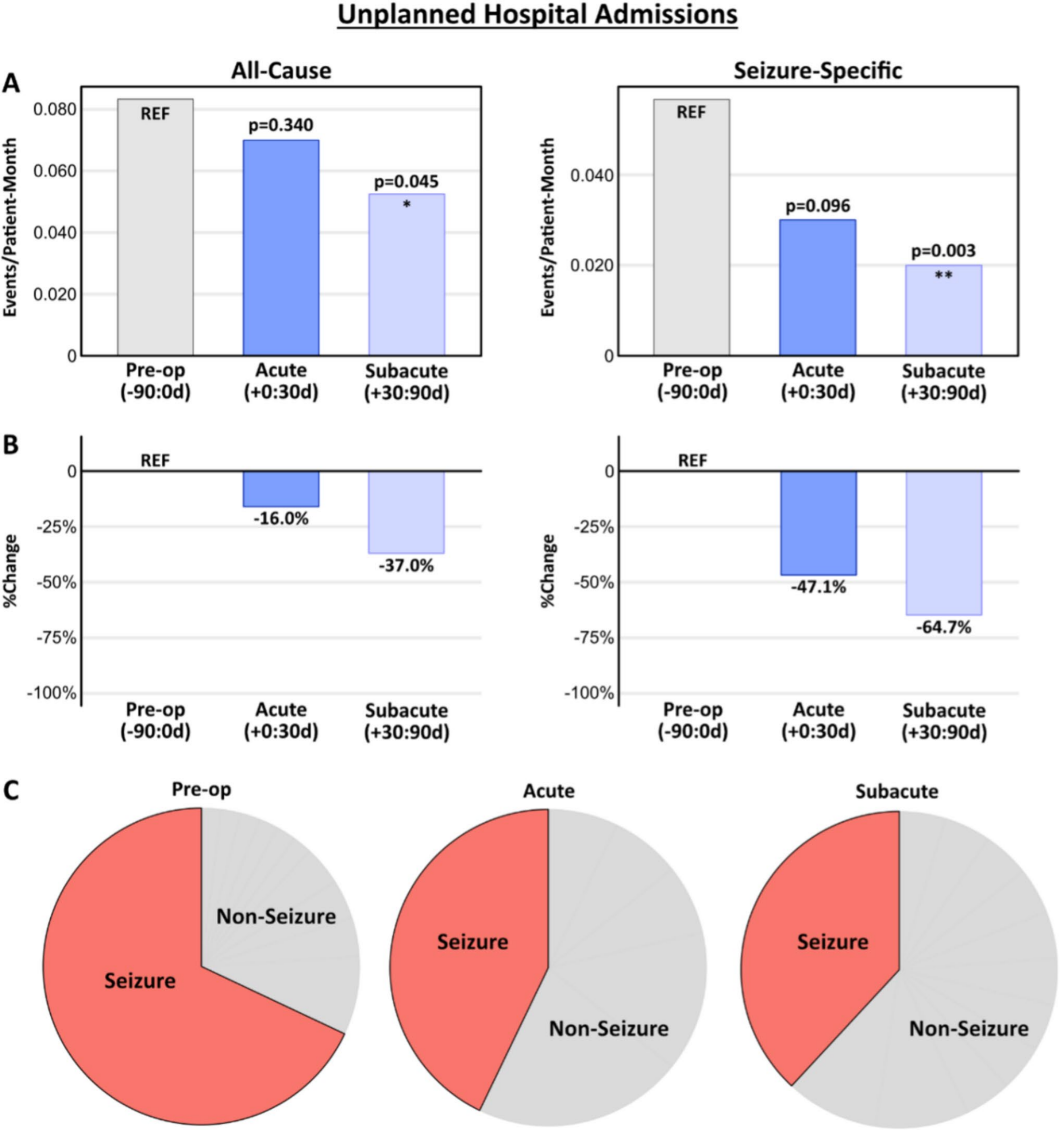
Unplanned hospital admissions following VNS implantation. Rates (**A**) and percent change (**B**) of unplanned hospitalizations for all-cause (left) and seizure-specific (right) indications across the study windows. Rates are presented as events per patient-month. *p*-values reflect a comparison to the baseline using a paired Poisson test. (**C)** Pie charts illustrate the breakdown of seizure versus non-seizure indications for unplanned admissions across the study windows, demonstrating a post-operative reduction in seizure-related presentations compared to baseline. Detailed pie charts with labeling for all non-seizure indications can be found in Supplemental Fig. S1

**Fig. 3:**
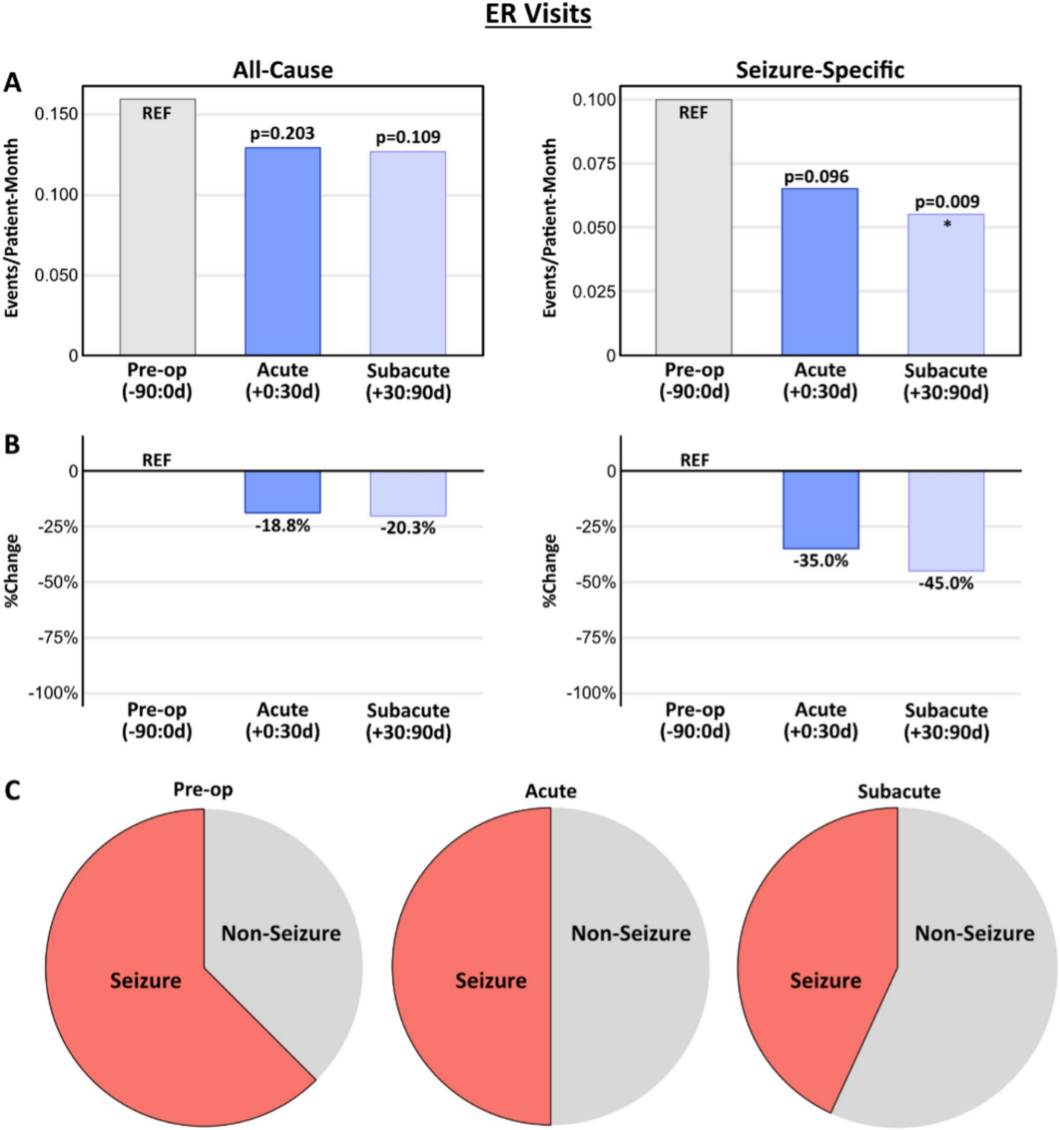
ER visits following VNS implantation. Rates (**A**) and percent change (**B**) of ER visits for all-cause (left) and seizure-specific (right) indications across the study windows. Rates are presented as events/patient-month. *p*-values reflect a comparison to the baseline using a paired Poisson test. (**C)** Pie charts illustrate the breakdown of seizure versus non-seizure indications for ER visits across the study windows, demonstrating a post-operative reduction in seizure-related presentations compared to baseline. Detailed pie charts with labeling for all non-seizure indications can be found in Supplemental Fig. S2

Rates and indications of unplanned acute-care peri-operative encounters are presented in Figs. 2 and 3. When unplanned hospital admissions were considered (Fig. 2), we found a significant reduction in the rate of admissions in the subacute post-operative window (0.0525 admissions/patient-month) compared to the pre-operative baseline window (0.0833 admissions/patient-month) (*p* = 0.045; Fig. 2a, b, left). When unplanned seizure-related admissions were analyzed separately, we observed a trend towards reduced rates of inpatient admissions in the acute post-operative window (−47.1%, *p* = 0.096), and a significant reduction in admission rate in the subacute window (−64.7%, *p* = 0.003) compared to baseline (Fig. 2a, b, right). In total, unplanned seizure-related admissions fell from 34 events in the 90 days before the procedure to 14 events combined across the acute and subacute post-operative windows, representing a 58.8% reduction from baseline.

Non-seizure indications for hospital admission included respiratory infections, GI symptoms, gastrostomy tube issues, and other non-VNS-related complaints (Fig. 2c; Supplemental Fig. S1). There were no acute-care presentations for surgical site infections or device malfunctions requiring revision. There were two admissions for VNS-related issues: one chest hematoma and one chest seroma, both self-limited. One patient developed non-infectious superficial wound separation requiring superficial wound revision as an outpatient.

### ER visits

When all-cause ER visits were examined, post-operative rates of ER visits were reduced, though the change was not statistically significant in either the acute or subacute post-operative window (Fig. 3a, b, left). Rates of seizure-related ER visits trended down in the acute post-operative window (−35.0%, *p* = 0.096) and were significantly reduced in the subacute window (−45.0%, *p* = 0.009). Total ER visits for seizures decreased from 60 to 35 over the full peri-operative window (41.7% reduction from baseline). Other non-seizure causes for ER visits included respiratory infections, GI symptoms, and issues with GI tubes or tracheostomies (Fig. 3c; Supplemental Fig. S2).

### Clinic assessments and patient communications

Procedure-related concerns documented within clinic notes and patient communications within 30 days of surgery are summarized in Table 2. The most frequent clinic complaints related to VNS surgery included stimulation-related effects (*n* = 41, 20.5%), including stimulation-induced hoarseness, cough, voice change, or vibration. Other complaints in this setting included pain (*n* = 6, 3.0%), incision or wound care concerns (*n* = 5, 2.5%), and post-operative hoarseness (*n* = 3, 1.5%). In telephone and electronic patient communications, the most frequently addressed issues were pain (*n* = 7, 3.5%), fever (*n* = 6, 3.0%), bruising or swelling of the surgical site (*n* = 5, 2.5%), and incision or wound care (*n* = 3, 1.5%).

**Table 2:** Comprehensive record of ambulatory visit complaints and telephone encounters within the 30-day period after VNS implantation

**Ambulatory office complaints**	*N *(%)
Stimulation-related effect	41 (20.5%)
Pain	6 (3.0%)
Incision/wound care	5 (2.5%)
Hoarseness	3 (1.5%)
Bruising/swelling/edema	2 (1.0%)
Cough/throat clearing	2 (1.0%)
Muscle spasm	2 (1.0%)
Mental status concern	2 (1.0%)
Bradycardia	1 (0.5%)
Dressing issue	1 (0.5%)
Halitosis	1 (0.5%)
Rash	1 (0.5%)
Seizure	1 (0.5%)
Seroma	1 (0.5%)
Sleep issue	1 (0.5%)
Voice change	1 (0.5%)
Vomiting	1 (0.5%)
Wound dehiscence	1 (0.5%)
**Telephone complaints**	
Pain	7 (3.5%)
Fever	6 (3.0%)
Bruising/swelling/edema	5 (2.5%)
Incision/wound care	3 (1.5%)
GI issue	3 (1.5%)
Dressing issue	2 (1.0%)
Rash	2 (1.0%)
Hoarseness	2 (1.0%)
Lethargy	2 (1.0%)
Sleep issue	2 (1.0%)
Bradycardia	2 (1.0%)
Stimulation-related effect	2 (1.0%)
Blushing	1 (0.5%)
Voice change	1 (0.5%)
Shortness of breath	1 (0.5%)
Cough/throat clearing	1 (0.5%)
Feeding issue	1 (0.5%)
Behavioral/mood issue	1 (0.5%)
Vomiting	1 (0.5%)
Seizure	1 (0.5%)

## Discussion

This study presents a large, single-surgeon series evaluating early unplanned healthcare utilization outcomes following VNS implantation in children with DRE. Our findings provide novel evidence for a significant reduction in seizure-related acute healthcare encounters shortly after VNS initiation.

A key and novel finding of this study is the rapid impact of VNS and the associated perioperative care model on reducing severe seizure events that require acute medical care. While the efficacy of VNS in reducing seizure frequency is typically evaluated over longer periods, such as 6 to 12 months, our data reveal a significant reduction in unplanned seizure-related hospital admissions and emergency visits within the first 3 months after surgery. Specifically, we observed a 59% reduction in seizure-related admissions and a 42% decrease in seizure-related ER visits in the 90 days following the procedure. This finding is particularly noteworthy because hospital utilization serves as an objective proxy for severe health events, which is less prone to the placebo effects that can influence subjective, parent-reported seizure outcomes [9]. Furthermore, we meticulously excluded any presentations for scheduled or elective purposes, such as phase 1 or 2 epilepsy evaluation, which otherwise would have inflated the rate of acute-care utilization during the pre-operative baseline window. To our knowledge, this is the first study to specifically isolate the immediate post-operative period (0–90 days) to quantify a reduction in severe seizure events during the initial VNS programming period. This early decrease in acute care needs can significantly improve a patient’s safety and quality of life, as well as lower healthcare costs, even before the full effects of seizure reduction are realized [10].

Our findings of an early reduction in healthcare provide important context to other recent studies. A 2024 study by Muthiah et al. [11] which analyzed a 2-year pre- versus 2-year post-operative window found no significant difference in the total number of seizure-related ED visits. Our study, however, focuses on a distinct and clinically critical 90 day post-operative period, which revealed a significant effect driven by mechanisms unique to the peri-operative phase. Given that VNS titration to therapeutic levels often takes months, it is essential to consider mechanisms for this 90-day effect, which are likely multifactorial. Following surgery, patients are more closely monitored through scheduled post-operative appointments with both the neurosurgery and neurology teams [1]. This increased ambulatory engagement provides families with a more structured support system and more frequent opportunities for in-person evaluation by a healthcare provider, perhaps reducing their reliance on emergency services [12]. The intervention and its associated programming may encourage a higher threshold for seeking emergency care. We acknowledge that the rapid titration protocol utilized in this study requires frequent outpatient visits, which may not be feasible in all practice settings depending on resource availability and geographic distance. Finally, we cannot entirely exclude a biological effect, driven by a transient “lesion effect” from surgical manipulation of the vagus nerve or subtle disruption of tract homeostasis.

The procedural complications noted in our study were generally minor and infrequent. Within the first 30 days, the most common complaints during clinic visits were related to the stimulation itself, such as hoarseness or coughing. The rare, surgery-related issues raised in phone calls and electronic messages were most often about pain, incision care, or swelling. Notably, there were no instances of surgical site infections or device malfunctions that required revision surgery within the entire 90-day post-operative study period.

Strengths of this study include the large cohort size and the detailed analysis of both surgical complications and early hospital utilization patterns.

## Conclusions

VNS implantation in children with DRE is associated with a significant and early reduction in seizure-related hospital admissions and emergency visits, which may serve as a proxy for severe seizure events. These findings are critical for physicians counseling families regarding the risks and benefits of VNS therapy, highlighting an important early positive impact on healthcare utilization in children with difficult-to-treat epilepsy.

## Supplementary Information

Below is the link to the electronic supplementary material.

ESM1 (DOCX 522 KB)

## Data Availability

No datasets were generated or analysed during the current study.

## Declarations

### Previous presentations

*Surgical Complications and Admission Rates After Vagus Nerve Stimulation in Children*, AANS 2025.
