# Supplementary material for: Early reduction in unplanned healthcare utilization following vagus nerve stimulation for pediatric epilepsy
Source: Childs Nerv Syst. 2026 May 8;42(1):206. doi: 10.1007/s00381-026-07245-5 (PMC13152911; doi:10.1007/s00381-026-07245-5)
Supplement: Supplementary file 1 — (DOCX 522 KB) [file 381_2026_7245_MOESM1_ESM.docx]

**Supplementary Materials**


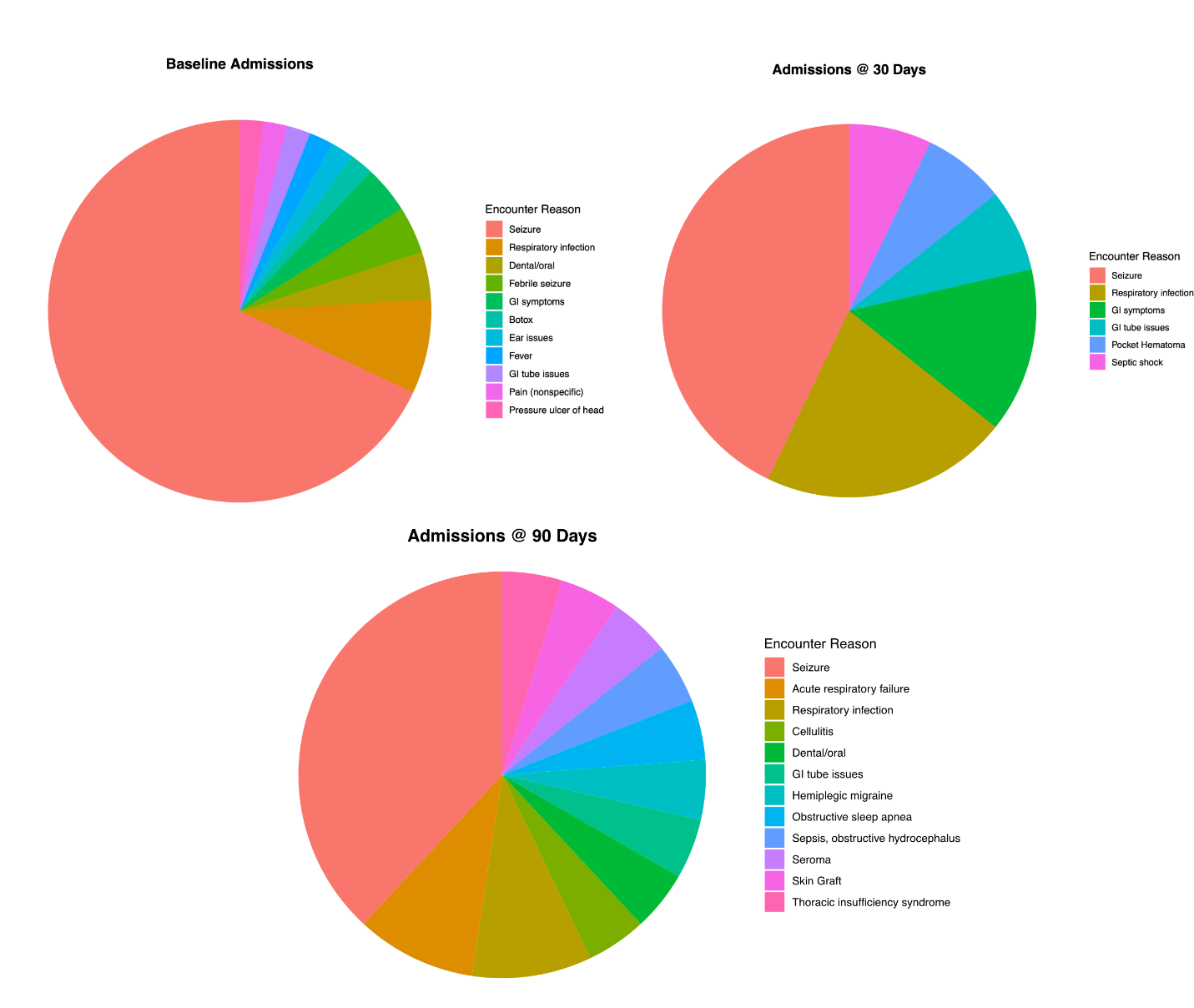


**Supplemental Figure S1.** Pie charts illustrate the breakdown of seizure versus non-seizure indications for unplanned admissions across the study windows, demonstrating a post-operative reduction in seizure-related presentations compared to baseline.


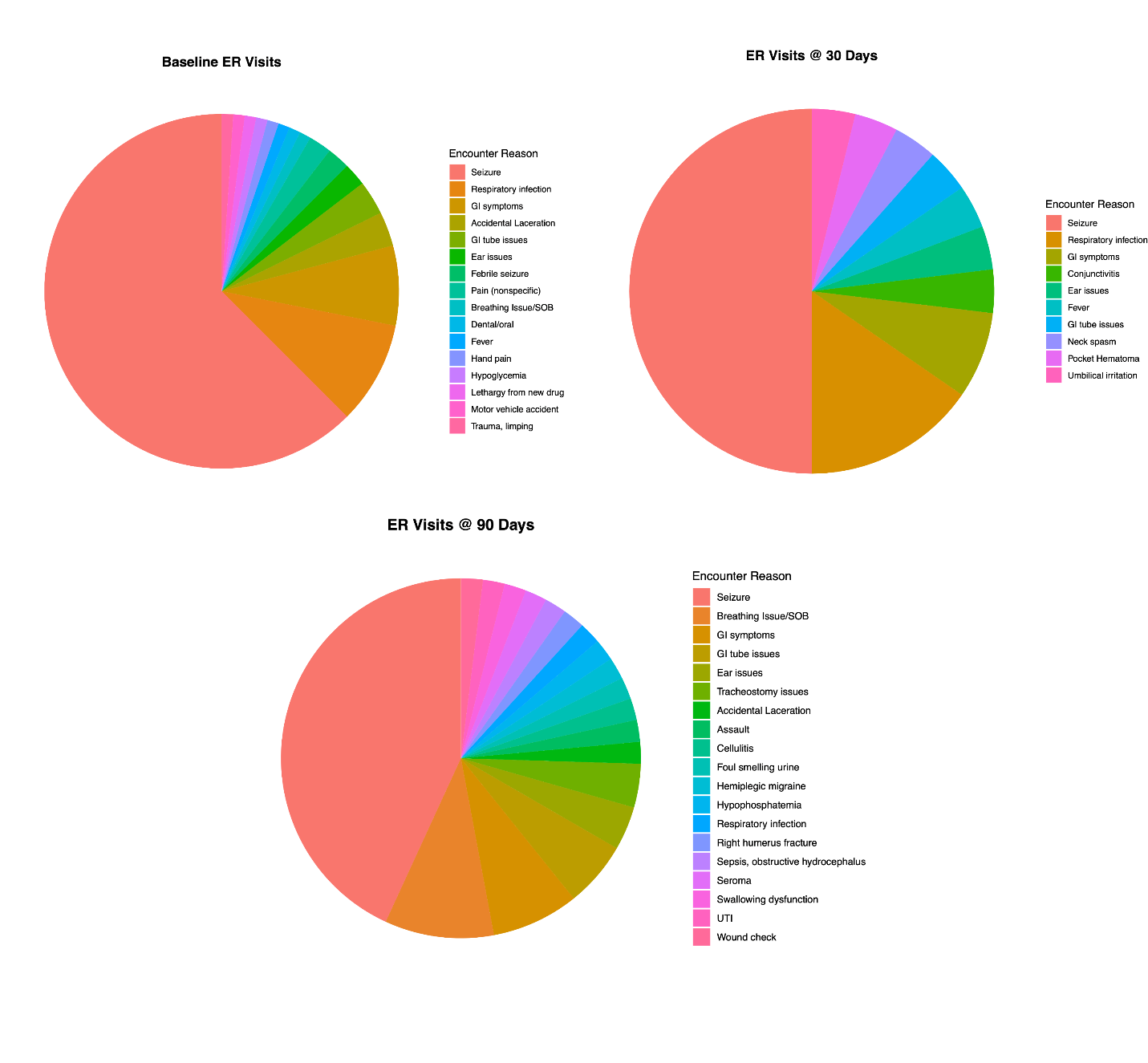


**Supplemental Figure S2.** Pie charts illustrate the breakdown of seizure versus non-seizure indications for ER visits across the study windows, demonstrating a post-operative reduction in seizure-related presentations compared to baseline.
